# Evaluation of Fluorescent Cu^2+^ Probes: Instant Sensing, Cell Permeable Recognition and Quantitative Detection

**DOI:** 10.3390/molecules26020512

**Published:** 2021-01-19

**Authors:** Hao He, Zhao Cheng, Lei Zheng, Xuejiao Zhang

**Affiliations:** School of Pharmacy, Xi’an Medical University, Xi’an 710021, China; hehao313@163.com (H.H.); zhengleixa@163.com (L.Z.); zhangxuejiao@xiyi.edu.cn (X.Z.)

**Keywords:** rhodamine, naked-eye recognition, copper ions, dynamic bioimaging, semiquantitative test paper

## Abstract

By incorporating a rhodamine spirolactam structure as the recognition site for Cu^2+^, two novel probes were synthesized through a connection of rhodamine 6G acylhydrazine and 5-formyl-6-hydroxyl-4-methylcoumarin/2,4-dihydroxybenzaldehyde. In the recognition process of probes towards Cu^2+^, the spirolactam ring exhibited opening and closing, accompanying an instant and specific change in fluorescence and in color, which could also achieve a naked-eye and semiquantitative recognition of aqueous Cu^2+^ besides the fluorescent Cu^2+^ detection method. Fluorescent analyses and ECV304 cell imaging further revealed the probes’ good optical stability, instant response, low toxicity, and membrane permeability, which offers future possibilities for the probes’ instant detection and the real-time tracking of Cu^2+^ in biological systems.

## 1. Introduction

As an abundantly existing element with the highest concentration occurring in the brain [1], copper [2,3] is an active participant in the human body and its abnormal changes have been proven to be connected with a number of neurodegenerative diseases [4,5]. On the one hand, there are commonly coexisting oxidation states of cuprous and copper ions in the biological systems, on the other hand, there are a variety of complex changes of copper, including the distribution, storage, and transportation, thus, if out of order [6], the dynamic changes of copper would induce abnormal accumulation or uncontrolled oxidation-reduction reactions [7], then disrupt the natural balance or trigger chain reactions, finally result in neurodegenerative diseases like Alzheimer’s and Parkinson’s Diseases [8]. In order to better understand the dynamic changes of copper, increasing copper-detecting techniques, especially those that could efficiently distinguish cuprous ions from copper ones without sample damages, need to be developed.

With numerous Cu^2+^-detecting methods [9,10] having been developed, the fluorescent probe method has been receiving much attention for the probes’ characteristics, such as tangible fluorescent signals [11] and instant responses to the target [12,13]. Especially, Cu^2+^ probes of rhodamine spirolactam-type, outstand for their remarkable copper ions assessment and specific color changes accompanied the recognition process. From the pioneering studies of rhodamine Cu^2+^ probes by A. Czarnik [14], to D. Wu [15] and P. Li [16], until recent research works by C. Liu [17] and M. Tian [18], increasing efforts were made to solve the remained problems in practical Cu^2+^ detections, such as enhancing the probes’ sensitivity and selectivity [19] towards Cu^2+^ in complex environments or biological systems, enforcing the probes’ long-wavelength excitation and emission characteristics [20] to avoid biological interferences, increasing the probes’ solubilities [15] and affinities [21], and so on.

**Figure 1:**
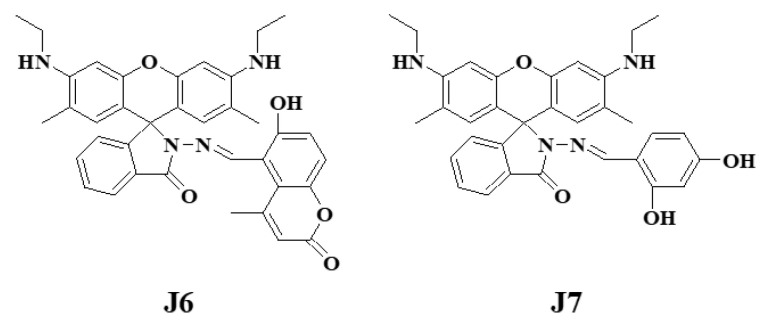
Structures of J6 and J7.

Accordingly, two novel Cu^2+^ probes were synthesized through a connection of rhodamine 6G moiety to a coumarin derivative (to obtain probe J6) and to a salicylaldehyde derivative (to obtain J7), respectively (Figure 1). The probes demonstrated specific responses to Cu^2+^ over other metals (Cu^+^ included) with remarkable spectral changes in both aqueous solutions and living cells. Apart from the highly qualitative Cu^2+^ detection, the synthesized two probes possessed good quantitative relationship and steady combination with Cu^2+^ in a relatively wide concentration range, not only at different pH values, but in time-lapse environments. Their good qualitative and quantitative relationships offer the probes a practical value for instant detection and time-lapse tracking of intracellular Cu^2+^ in biological systems. Additionally, semiquantitative Cu^2+^ test paper was prepared based on the probes’ naked-eye recognition characteristics, giving us a quick and convenient Cu^2+^ colorimetric method.

## 2. Results

### 2.1. Structure Characterization

By introducing coumarin and salicylaldehyde derivatives to a rhodamine 6G moiety, two novel fluorescent probes J6 and J7 for Cu^2+^ were synthesized. The structures of J6 and J7 were characterized by IR, ^1^H NMR and MS. The calculation and measurement results of MS were all matched, ^1^H NMR chemical shifts were in accordance with the target structures, and the characteristic absorbance peaks of newly synthesized functional groups C=O and C=N, both located at the spirolactam ring, were at the reasonable ranges of ~1700 and 1600~1620 cm^−1^ in IR spectra.

### 2.2. Metal Ion Selectivity

**Figure 2:**
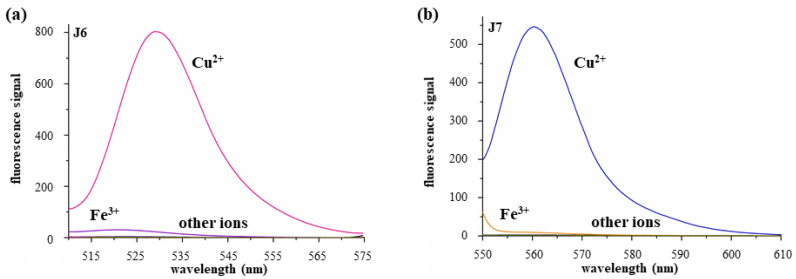
Fluorescence intensity changes of (**a**) J6 and (**b**) J7 upon the addition of various metal ions.

In biological systems, the complex coexistence of multiple ions is making the task of a specific detection on the target ion much more difficult. And high selectivity has been therefore becoming a very important evaluation parameter concerning the probes’ performance. By incorporating the spirolactam ring with an opening and closing transformation, the synthesized rhodamine probes J6 and J7 showed highly critical responses towards Cu^2+^. When excited by the wavelength of 470 nm, neither probe J6 itself nor the coexistence of J6 and other ions except Cu^2+^ displayed obvious fluorescence emission at 529 nm, indicating a predominantly closed spirolactam form in J6. Otherwise, the addition of Cu^2+^ could immediately trigger a significant fluorescence intensity enhancement at 529 nm (Figure 2a), only with a relatively low interference coming from Fe^3+^, which suggested the opening of initially closed spirolactam ring in the recognition process of J6 to Cu^2+^. A similar phenomenon was observed in J7 and the fluorescence increase of J7-Cu^2+^ emerged at 560 nm when excited by 507 nm (Figure 2b).

**Figure 3:**
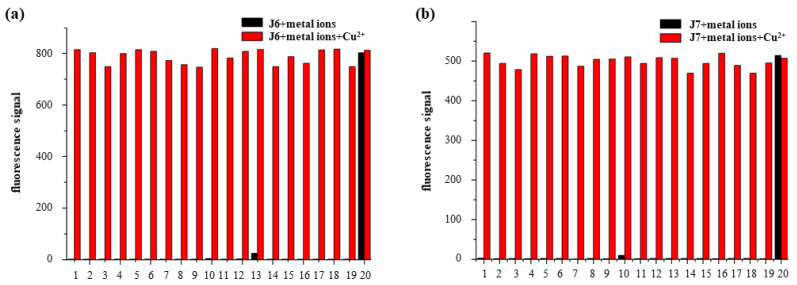
Selectivity of (**a**) J6 and (**b**) J7 to Cu^2+^ in Cu^2+^-competing ions coexisting system with mixed solvents (*V* (EtOH): *V* (H_2_O) = 5: 5, Tris-HCl pH = 7.4) environment. (**a**) J6: from left to right (bar 1-20): K^+^, Na^+^, Ca^2+^, Mg^2+^, Ba^2+^, Al^3+^, Ni^2+^, Pb^2+^, Mn^2+^, Cr^3+^, Co^2+^, Ag^+^, Fe^3+^, Zn^2+^, Cu^+^, Fe^2+^, Cd^2+^, Sn^2+^, Hg^2+^ and Cu^2+^. (**b**) J7: from left to right (bar 1-20): K^+^, Na^+,^ Ca^2+^, Mg^2+^, Ba^2+^, Al^3+^, Ni^2+^, Pb^2+^, Mn^2+^, Fe^3+^, Cr^3+^, Co^2+^, Ag^+^, Zn^2+^, Cu^+^, Fe^2+^, Cd^2+^, Sn^2+^, Hg^2+^ and Cu^2+^.

In the presence of other metal ions, experiments on ions competition were conducted by adding Cu^2+^ to the solution of probe J6 (J6: 10 μmol L^−1^, metal ions: 10 μmol L^−1^) at an excited wavelength of 470 nm. The spectral enhancement induced by Cu^2+^ at 529 nm, evidently, was almost not affected by the presence of other metal ions (Figure 3a). The ions competition tests indicated that probe J6 was highly sensitive towards Cu^2+^ and provide a future use of J6 for Cu^2+^ detection in complex environments. Similar phenomena were observed at 560 nm for J7 when excited by 507 nm (Figure 3b).

### 2.3. Visual Detection of Copper Ions

**Figure 4:**
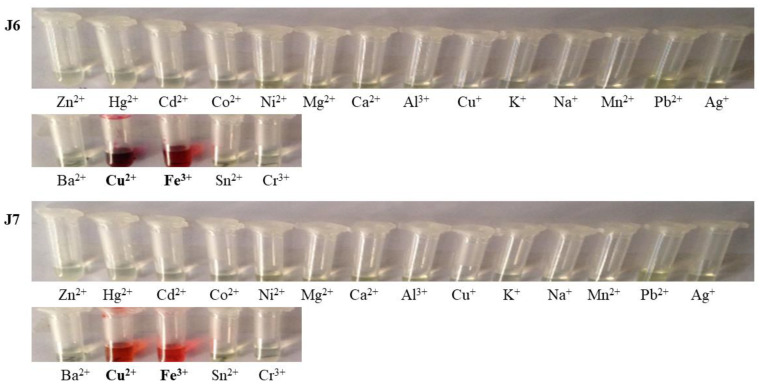
Visible color changes of J6 (**top**) and J7 (**bottom**) after the addition of Cu^2+^.

During the detections of Cu^2+^ by J6/J7, a significant color change, accompanying the fluorescence enhancement, emerged immediately upon the adding of Cu^2+^ to the probe, with low interferences of Fe^3+^. Instead, other metal ions’ addition did not induce any color changes (Figure 4).

Before and after Cu^2+^ addition, the J6/J7 solution exhibited an obvious and rhodamine-characteristic color change from colorless J6/J7 to the rhodamine red with an open spirolactam ring in J6/J7-Cu^2+^, which implied that J6/J7 showed a high selectivity for Cu^2+^ and could serve as “naked-eye” probes.

### 2.4. Quantitative Detection of Copper Ions

**Figure 5:**
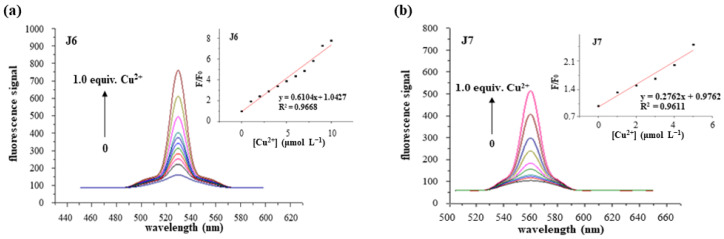
Fluorescence titration curve of (**a**) J6 and (**b**) J7 with Cu^2+^ (J6: 10 μmol L^−1^, J7: 10 μmol L^−1^).

To analyze the quantitative relationship of J6/J7 and Cu^2+^, fluorescence titration was performed. As shown in Figure 5a of J6 and Figure 5b of J7, probe J6 itself with a predominant spirolactam form displayed nearly no fluorescence at 529 nm, while addition of Cu^2+^ immediately induced opening of the initially closed rhodamine-spirolactam ring, accompanied by a significant fluorescence enhancement at 529 nm. To furtherly analyze the 529 nm fluorescence intensity of J6-Cu^2+^ system, a linear relationship between fluorescence intensity and concentration of Cu^2+^ was obtained, indicating a highly quantitative detection of Cu^2+^ by J6 in the concentration range of 0~10 μmol L^−1^ Cu^2+^ (Figure 5a, inset). The linear response of fluorescence emission intensity at 529 nm towards [Cu^2+^] in the range of 0~10 μmol L^−1^ could be expressed by the following Stern-Volmer Equation [22,23] through an analysis on the relationship of F/F_0_ (F: fluorescence intensity of the probe-Cu^2+^ system, F_0_: fluorescence intensity of the probe) versus [Cu^2+^]: y = 0.6104x+1.0427 (R^2^ = 0.9668). The association constant was *K*_a_ = 1.64 × 10^6^ L mol^−1^, and the corresponding detection limit of Cu^2+^ by J6 was calculated to be 0.592 μmol L^−1^.

**Figure 6:**
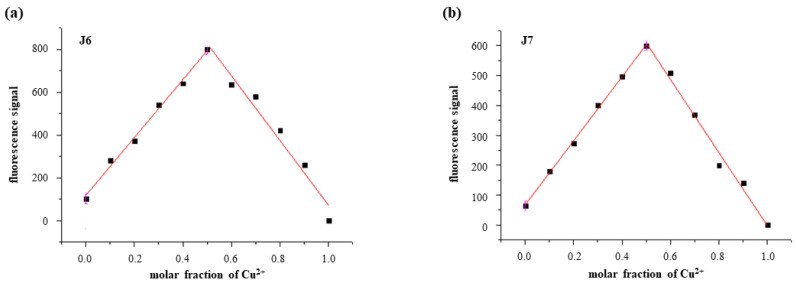
Association stoichiometry of (**a**) J6-Cu^2+^ and (**b**) J7-Cu^2+^.

A stoichiometry calculation was performed to better understand the probes’ sensitivity, indicating a 1:1 stoichiometry between J6 and Cu^2+^ was adopted, as shown in Figure 6a.

As for J7, the linear relationship at 560 nm towards [Cu^2+^] in the range of 0~6 μmol L^−1^ (Figure 5b, inset) based on Stern-Volmer Equation could be expressed as y = 0.2762x + 0.9762 (R^2^ = 0.9611), with a Cu^2+^ detection limit of 0.977 μmol L^−1^ and the association constant *K*_a_ as 3.62 × 10^6^ L mol^−1^, also demonstrating 1:1 association stoichiometry between J7 and Cu^2+^ (Figure 6b).

**Figure 7:**
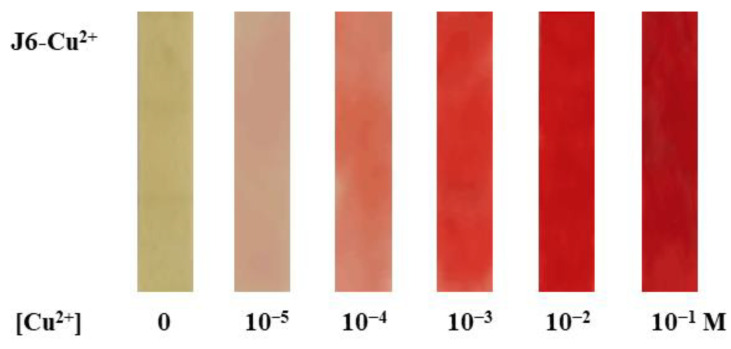
Semiquantitative test paper for Cu^2+^ by J7.

A semiquantitative test paper was prepared for a compatible detection of Cu^2+^, the characteristic rhodamine red of an opening spirolactam ring exhibited a positive correlation with [Cu^2+^] through gradient color changes (Figure 7), which could be distinguished by the naked eye, affording a quick and convenient Cu^2+^ semiquantitative colorimetric method.

### 2.5. Influences of pH and Time on the Detection of Copper Ions

Considering the complex environment in biological applications of the probes, the fluorescence stability at physiological pH [24] was examined. At a varied pH region of 5.4 to 7.4, fluorescence intensity of both the J6/J7 and J6/J7-Cu^2+^ systems kept steady (Figure S1), suggesting a relatively wide pH range from 5.4 to 7.4 appropriate for J6/J7-Cu^2+^ combination system.

As for time influence on the fluorescence intensity of probe-Cu^2+^ system, the J6/J7-Cu^2+^ remained steady for hours (Figure S2), indicating a possible time-lapse imaging application apart from an instant fluorescence imaging.

### 2.6. MTT Assay

**Figure 8:**
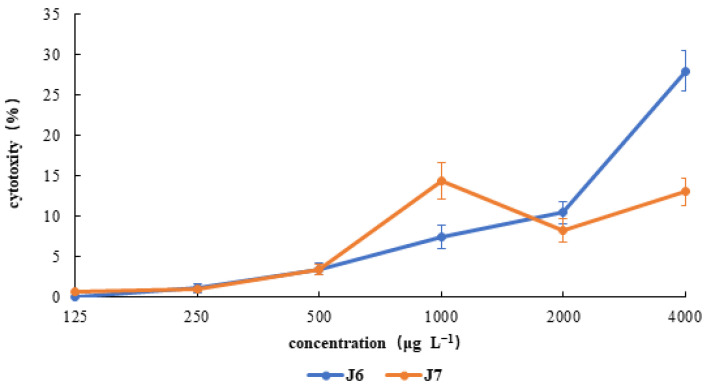
The cytotoxicity of J6/J7 on ECV304 at 24 h.

To evaluate the potential imaging and diagnostic applications [15] of the two probes J6 and J7, MTT assay was performed in ECV304 cells with a concentration gradient of 125, 250, 500, 1000, 2000 and 4000 μg L^−1^ for each probe. The cytotoxicity of J6 and J7 in ECV304 cells at 24 h were compiled in Table S1 and depicted in Figure 8, indicating that there was no significant toxic effect of J6 and J7 on ECV304 cells when incubated for 24 h. Even when the concentration of J6/J7 reached the highest 4000 μg L^−1^ in the assay, the cytotoxicity of ECV304 cells remained below 30%, indicating a relatively low cytotoxicity of J6/J7 in the range of 125~4000 μg L^−1^, which satisfies the cytotoxicity-concentration demands of μmol L^−1^ Cu^2+^ distributions in most biological systems.

### 2.7. Recognition Mechanism

**Figure 9:**
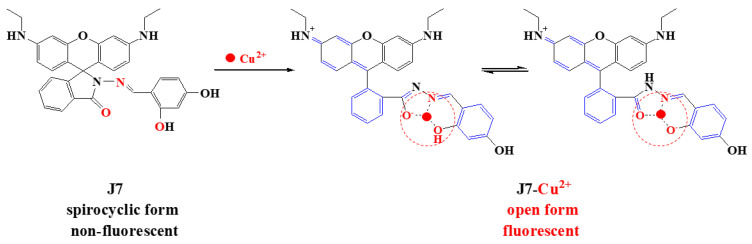
Recognition mechanism of the probe to Cu^2+^ (shown as J7).

Besides a structural transformation of the spirolactam ring from initially closed to later opening in the recognition process of probe-Cu^2+^, fluorescence increase at the target emission wavelength could also be attributed to a possible structural cavity [15,25] to capture Cu^2+^ (Figure 9) and electron effect [26].

In J6/J7, the two oxygen and one nitrogen atoms located around the spirolactam ring, with higher nucleophilic activity towards Cu^2+^, probably act as the recognition sites to capture target Cu^2+^. When the spirolactam ring opens and thus the three atoms form an appropriate cavity to hold Cu^2+^, then the unbonding electron pairs of the two oxygen and one nitrogen atoms will partly be provided to fulfill the empty electron orbits of Cu^2+^ through intramolecular forces [27,28], though weaker than coordination bonds, an obvious electron transfer [29] will then be established and two chelating five- and six-membered rings formed, which contributes to a better planarity and rigid of the J6/J7-Cu^2+^ system. And stronger fluorescence intensity of the J6/J7-Cu^2+^ system accompanied with the extended conjugated chain (the extended conjugation chain was labelled in blue in Figure 9) and better molecular planarity.

Unlike the reaction-based irreversible chemosensors for copper (II), probes J6/J7 bind reversibly to Cu^2+^ due to their multidentate chelation model [30] in the recognition mechanism and could be better applied to Cu^2+^ detections in mild-reaction needed biological environments.

### 2.8. Bioimaging

**Figure 10:**
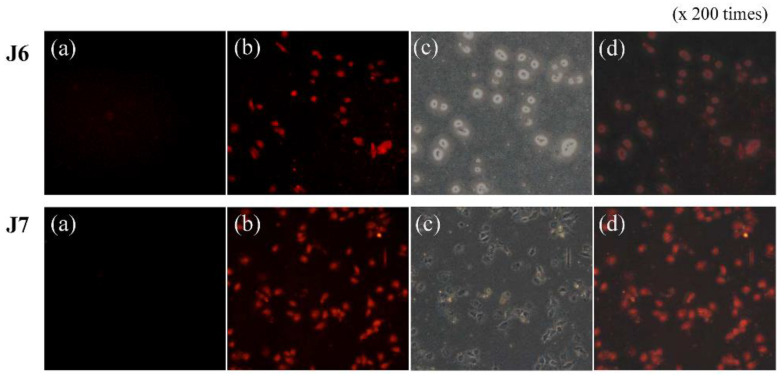
(**a**) Fluorescent images of ECV304 incubated with 10 μmol L^−1^ J6/J7 for 30 min; (**b**) a further incubation with 10 μmol L^−1^ copper ions for 2 h; (**c**) bright-field image of (**b**); (**d**) an overlay image of (**b**,**c**).

After an initial incubation of ECV304 and J6/J7 (Figure 10a), 10 μmol L^−1^ Cu^2+^ was added in, incubated for another 2 h at 37 °C, and red fluorescence was emitted from the intracellular area (Figure 10b) in comparison with Figure 10a, demonstrating the recognition process of added Cu^2+^ by J6/J7 accomplished almost instantly. The overlay of fluorescence image (Figure 10b) and bright-field transmission image (Figure 10c) revealed that the fluorescence signals were from the cytosol [31,32] (Figure 10d), which indicated the good cell permeability of J6/J7 and the future applications of J6/J7 for dynamic imaging and tracking of Cu^2+^ in living cells.

## 3. Discussion

Novel rhodamine-based Cu^2+^ probes J6 and J7 were synthesized, with their structures characterized by IR, MS, and ^1^H NMR, and biological applications analyzed by FS, MTT, and bioimaging. J6 and J7 demonstrated high selectivity to Cu^2+^ over other metal ions and could be used for highly specific recognition of Cu^2+^ in ions coexisting environment. Accompanying the recognition process was a significant color change, which enabled the “naked-eye” detection of aqueous Cu^2+^ and was prepared as a compatible and semiquantitative Cu^2+^ test paper. Further fluorescence titration and stoichiometry revealed the detection limits of Cu^2+^ by J6/J7 to be 0.592 and 0.977 μmol L^−1^, although not as good as the 10^−8^ mol L^−1^ concentration limit seen in some commercial Cu^2+^ probes, the LOD value could still satisfy the demands of μmol L^−1^ Cu^2+^ distributions in most biological systems. Low cytotoxicity and instant signaling in fluorescence imaging of Cu^2+^ in living cells ECV304 suggested the probe’s future applications as an instant Cu^2+^ detection method in clinical diagnosis and a dynamic tracking tool for Cu^2+^ in biological systems.

## 4. Materials and Methods

### 4.1. Materials and Instrumentation

All the reagents and solvents used for synthesis were analytically pure and used without further treatment unless otherwise noted. The ECV304 cells were purchased from KeyGen BioTECH (Nanjing, China). The reaction process was monitored by thin-layer chromatography (TLC) on silica gel GF254. The products were purified by column chromatography on Merck silica gel (250–400 mesh ASTM). A Tris-HCl buffer solution (pH 7.4) was prepared using 0.1 mol L^−1^ HCl and proper amount of a 0.1 mol L^−1^ Tris stock solution (Sinopharm Chemical Reagent Company, Shanghai, China). Double distilled water was used throughout the process of solution preparing and spectroscopic testing. Metal ion solutions were prepared from their nitrate and chloride salts, KCl, NaCl, AgNO_3_, Ca(NO_3_)_2_·4H_2_O, Mg(NO_3_)_2_·6H_2_O, BaCl_2_, NiCl_2_, PbCl_2_, MnCl_2_, CoCl_2_, Zn(NO_3_)_2_·6H_2_O, Cd(NO_3_)_2_·2H_2_O, SnCl_2_, HgCl_2_, AlCl_3_, Cr(NO_3_)_3_·9H_2_O, FeCl_2_, Fe(NO_3_)_3_·9H_2_O, CuCl, and Cu(NO_3_)_2_·3H_2_O, respectively.

Fourier Transform-Infrared (FT-IR) spectra were recorded with KBr pellets on a Bruker EQUINOX-55 FT-IR spectrometer (Karlsruhe, Germany). Elemental Analyses (EA) were recorded on an Elementar varioEL III analyzer. ^1^H Nuclear Magnetic Resonance (^1^H NMR) spectra were recorded on a Varian INOVA-400 spectrometer (Palo Alto, CA, USA) at 400 MHz and chemical shifts were reported relative to the internal standard tetramethylsilane (TMS). Fluorescence spectra were recorded on a HITACHI F-4500 fluorescence spectrophotometer (Tokyo, Japan). Mass Spectrometry (MS) analyses were performed using an Agilent 1260-6460A triple quadrupole liquid chromatograph-mass spectrometer (Santa Clara, CA, USA). The cytotoxicity results were analyzed with the SoftMax Pro Software (version 2.2.1) (San Jose, CA, USA) in a Molecular Devices SpectraMax 190 microplate reader (San Jose, CA, USA). The cell-imaging experiments were performed using an OLYMPUS U-LH 100HG IX73 fluorescence microscope.

### 4.2. Synthesis and Characterization of Probes J6/J7

**Scheme 1:**
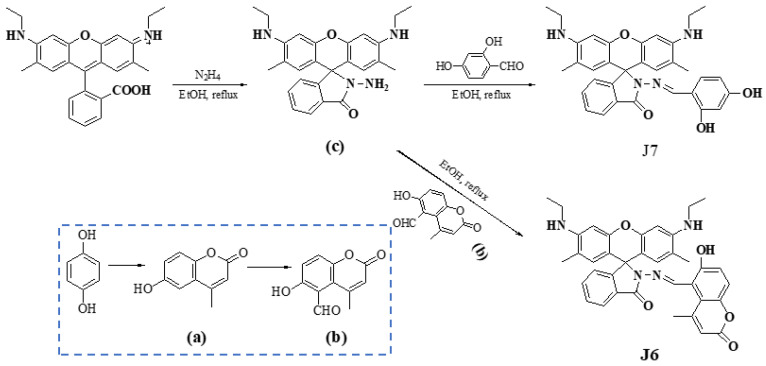
Synthetic routes of J6 and J7. (**a**) 6-Hydroxyl-4-methylcoumarin, (**b**) 5-formyl-6-hydroxyl-4-methylcoumarin, (**c**) rhodamine 6G acylhydrazine

Target probes J6 and J7 were synthesized in a route shown in Scheme 1. The structures of J6 and J7 were characterized by IR, ^1^H NMR, and MS.

#### 4.2.1. 6-Hydroxyl-4-Methylcoumarin (Scheme 1a)

With the temperature controlled between 0 and 5 °C in an ice bath, *p*-benzenediol (5.0 g, 0.045 mol) and ethyl acetoacetate (15 mL, 0.12 mol) were first added to concentrated sulfuric acid (25 mL), then the mixture was kept between 0 and 5 °C and stirred for 24 h, poured into iced water (100 mL), filtered, and subsequently purified by column chromatography (silica gel, *V* (EtOAc): *V* (hexane) = 1: 2 as the eluent) to obtain 6-hydroxyl-4-methylcoumarin (a).

6-Hydroxyl-4-methylcoumarin: yellow solid, 5.2 g, yield 75%. IR (KBr pellet, υ/cm^−1^): 3342 (υ_–OH_); 1691 (υ_C=O_); 1580 (υ_C=C_); 1443 (σ_–OH_); 1232 (υ_C–O–C_) (Figure S3). EA: C 68.17%, H 4.55% (calcd. for C_10_H_8_O_3_: C 68.18%, H 4.58%).

#### 4.2.2. 5-Formyl-6-Hydroxyl-4-Methylcoumarin (Scheme 1b)

With the temperature controlled between 0 and 5 °C in an ice bath, 6-hydroxyl-4-methylcoumarin (1.0 g, 5.0 mmol) and hexamethylenetetramine (2.0 g, 10 mmol) were added to trifluoroacetic acid (10 mL), the mixture was then refluxed at 70 °C for 10 h, monitored by TLC, cooled down, concentrated in vacuo, poured into iced water (50 mL), filtered, and subsequently purified by column chromatography (silica gel, *V* (EtOAc):*V* (hexane) = 1:2 as the eluent) to afford 5-formyl-6-hydroxyl-4-methylcoumarin (b).

5-Formyl-6-hydroxyl-4-methylcoumarin: yellow powder, 0.82 g, yield 63%. IR (KBr pellet, υ/cm^−1^): 3423 (υ_–OH_); 2800, 2700 (υ_C–H_); 1606 (υ_C=C_); 1367 (σ_–OH_) (Figure S4). EA: C 64.74%, H 3.97% (calcd. for C_11_H_8_O_4_: C 64.71%, H 3.95%).

#### 4.2.3. Rhodamine 6G Acylhydrazine (Scheme 1c)

At r. t., 98% hydrazine hydrate (3.5 mL, 0.069 mol) was added to a 20 mL ethanol solution of rhodamine 6G (3.0 g, 7.2 mmol); after that, the reaction mixture was refluxed at 80 °C for 3 h, monitored by TLC, then cooled down, filtered and washed with anhydrous ethanol, affording rhodamine 6G acylhydrazine (c).

Rhodamine 6G acylhydrazine: pink solid, 0.76 g, yield 75%. IR (KBr pellet, υ/cm^−1^): 3427 (υ_–NH2_); 1688 (υ_C=O_); 2926 (υ_C–H_), 1421 (σ_C–H_); 1623, 1516 (υ_C=C_) (Figure S5). EA: C 72.89%, H 6.57%, N 13.08% (calcd. for C_26_H_28_N_4_O_2_: C 72.87%, H 6.59%, N 13.07%).

#### 4.2.4. Probe J6

The synthesized rhodamine 6G acylhydrazine (0.17 g, 0.40 mmol) and 5-formyl-6-hydroxyl-4-methylcoumarin (0.10 g, 0.50 mmol) in previous steps were dissolved in anhydrous methanol (20 mL) and then refluxed at 65 °C for 1.5 h, monitored by TLC, cooled down, filtered, washed with anhydrous methanol, concentrated in vacuo, and subsequently purified by column chromatography (silica gel, *V* (EtOAc):*V* (hexane) = 1:1 as the eluent) to afford J6.

J6: yellow solid, 0.13 g, yield 50%. IR (KBr pellet, υ/cm^−1^): 3432 (υ_–OH_); 1726 (υ_C=O_); 1621 (υ_C=N_); 1516 (υ_C=C_); 1422 (σ_–OH_); 1216 (υ_C–O–C_) (Figure S6). ^1^H-NMR (400 MHz, CDCl_3_) δ: 10.89 (s, 2H, 2×–NH–), 8.93 (s, 1H, –OH), 8.06~8.01 (m, 2H, Ar-H), 7.57~7.48 (m, 4H, Ar-H), 7.19~7.14 (m, 2H, Ar-H), 7.03 (s, 2H, Ar-H), 7.01 (s, 2H, 2×=CH–), 2.02 (s, 4H, 2×–CH_2_–), 1.92 (s, 15H, 5×–CH_3_) (Figure S7). MS: *m/z* = 614.29 (calcd. for C_37_H_34_N_4_O_5_: 614.25) (Figure S8).

#### 4.2.5. Probe J7

Rhodamine 6G acylhydrazine (0.28 g, 0.60 mmol) and 2,4-dihydroxybenzaldehyde (0.12 g, 0.80 mmol) were initially dissolved in anhydrous methanol (20 mL) and then refluxed at 65 °C for 2 h, monitored by TLC, cooled down, filtered, washed with anhydrous methanol, concentrated in vacuo, and subsequently purified by column chromatography (silica gel, *V* (EtOAc):*V* (hexane) = 1:1 as the eluent) to afford J7.

J7: pink solid, 0.20 g, yield 47%. IR (KBr pellet, υ/cm^−1^): 3427, 3404 (υ_–OH_); 1673 (υ_C=O_); 1624 (υ_C=N_); 1518 (υ_C=C_); 1423 (σ_OH_); 1222 (υ_C–O–C_) (Figure S9). ^1^H-NMR (400 MHz, CDCl_3_) δ: 11.07 (s, 2H, 2×–NH–), 9.09 (s, 2H, 2×–OH), 8.00 (dd, *J* = 5.8, 2.6 Hz, 3H, Ar-H), 7.54~7.48 (m, 4H, Ar-H), 7.13 (dd, *J* = 5.7, 2.6 Hz, 3H, Ar-H), 6.95 (s, 1H, Ar-H), 6.93 (s, 1H, =CH–), 2.03 (s, 4H, 2×–CH_2_–), 1.93 (s, 12H, 4×–CH_3_) (Figure S10). MS: *m/z* = 548.29 (calcd. for C_33_H_32_N_4_O_4_: 548.24) (Figure S11).

### 4.3. Spectroscopic Analysis

Stock solutions (200 μmol L^−1^) of probe J6/J7, K^+^, Na^+^, Ag^+^, Ca^2+^, Mg^2+^, Ba^2+^, Ni^2+^, Pb^2+^, Mn^2+^, Co^2+^, Zn^2+^, Cd^2+^, Sn^2+^, Hg^2+^, Al^3+^, Cr^3+^, Fe^2+^, Fe^3+^, Cu^+^ and Cu^2+^ were prepared in a mixed solvent system (*V* (EtOH):*V* (H_2_O) = 5:5, Tris-HCl pH = 7.4) [33]. When used for fluorescence tests, the stock solutions were usually diluted to 10 μmol L^−1^ with mixed EtOH-H_2_O solvent (5:5, *V*/*V*, Tris-HCl pH = 7.4) unless otherwise noted. All the spectroscopic measurements were performed at least in triplicate and averaged.

### 4.4. Cytotoxicity

The cytotoxicity of J6 and J7 in ECV304 cells was analyzed by Methyl Thiazolyl Tetrazolium (MTT) assay [34]. ECV304 cells were cultured in Dulbecco’s Modified Eagle Medium (DMEM) with 10% Fetal Bovine Serum (FBS) in a humidified atmosphere with 5% CO_2_ at 37 °C. All cells in the exponential phase of growth were used in the experiments. After digestion with a 0.25% trypsin solution, the cells were seeded in 96-well cell culture clusters with 200 μL per well at a density of 2.5 × 10^4^ cells/mL for 24 h. The probes J6/J7 (100 mmol L^−1^ in DMSO) were then added to the 96-well plate to achieve a concentration gradient of 125, 250, 500, 1000, 2000, and 4000 μg L^−1^, and then incubated for another 24 h. Subsequently, the medium was removed, the cells were washed gently with Phosphate Buffered Saline (PBS) three times and incubated with 5.0 mg mL^−1^ MTT solution at 37 °C for 4 h. After that, the cells were washed gently with PBS three times, 150 μL DMSO was added to each well to dissolve the resulting crystals. The optical density was measured at 490 nm on a microplate spectrophotometer. All of the tests were conducted in triplicate. Data were expressed as mean ± standard deviation (SD).

### 4.5. Fluorescent Imaging in Living Cells

ECV304 cells were dispensed and cultured for 2 h until they plated on glass-bottomed dishes. The medium was then removed, and the cells were washed with DMEM and incubated for 30 min with 10 μmol L^−1^ of the probes at 37 °C, then washed three times with PBS and imaged [35]. After that, the cells were supplemented with 10 μmol L^−1^ of Cu^2+^ in the growth medium for another 30 min at 37 °C, washed three times with PBS, and imaged.

## Data Availability

The data presented in this study are available in the article and supplementary material.

## Supplementary Materials

The following are available online, Figure S1: Influence of pH on J6/J7 (8 μmol L^−1^) in the absence and presence of Cu^2+^ (8 μmol L^−1^), Figure S2: Influence of time on J6/J7-copper ions system (probe: 8 μmol L^−1^, Cu^2+^: 8 μmol L^−1^), Figure S3: IR of 6-hydroxyl-4-methylcoumarin (a), Figure S4: IR of 5-formyl-6-hydroxyl-4-methylcoumarin (b), Figure S5: IR of rhodamine 6G acylhydrazine (c), Figure S6: IR of probe J6, Figure S7: ^1^H NMR of probe J6, Figure S8: MS of probe J6, Figure S9: IR of probe J7, Figure S10: ^1^H NMR of probe J7, Figure S11: MS of probe J7, Table S1: Cytotoxicity of probes J6 and J7 in ECV304 at 24 h.

## Sample Availability

Samples of the compounds are available from the authors.
