# Supplementary material for: Evaluation of Fluorescent Cu2+ Probes: Instant Sensing, Cell Permeable Recognition and Quantitative Detection
Source: Molecules. 2021 Jan 19;26(2):512. doi: 10.3390/molecules26020512 (PMC7835809; doi:10.3390/molecules26020512)
Supplement: Supplementary file 1 [file molecules-26-00512-s001.pdf]

Supplementary Information

Evaluation of fluorescent Cu^{2+} probes: instant sensing, cell permeable recognition and quantitative detection

Hao He, Zhao Cheng*, Lei Zheng, Xuejiao Zhang

School of Pharmacy, Xi'an Medical University, 710021 Xi'an, PR China; hehao313@163.com (H.H.); zhengleixa@163.com (L.Z.); zhangxuejiao@xiyi.edu.cn (X.Z.)

* Correspondence: chengzhao@xiyi.edu.cn (Z.C.)

Contents:

Figure S1. Influence of pH on J6/J7 (8 μmol L^{-1}) in the absence and presence of Cu^{2+} (8 μmol L^{-1}).

Figure S2. Influence of time on J6/J7-copper ions system (probe: 8 μmol L^{-1}, Cu^{2+}: 8 μmol L^{-1}).

Table S1. Cytotoxicity of probes J6 and J7 in ECV304 at 24 h.

Figure S3. IR of 6-hydroxyl-4-methylcoumarin (a).

Figure S4. IR of 5-formyl-6-hydroxyl-4-methylcoumarin (b).

Figure S5. IR of rhodamine 6G acylhydrazine (c).

Figure S6. IR of probe **J6**.

Figure S7. ^{1}H NMR of probe **J6**.

Figure S8. MS of probe **J6**.

Figure S9. IR of probe **J7**.

Figure S10. ^{1}H NMR of probe **J7**.

Figure S11. MS of probe **J7**.

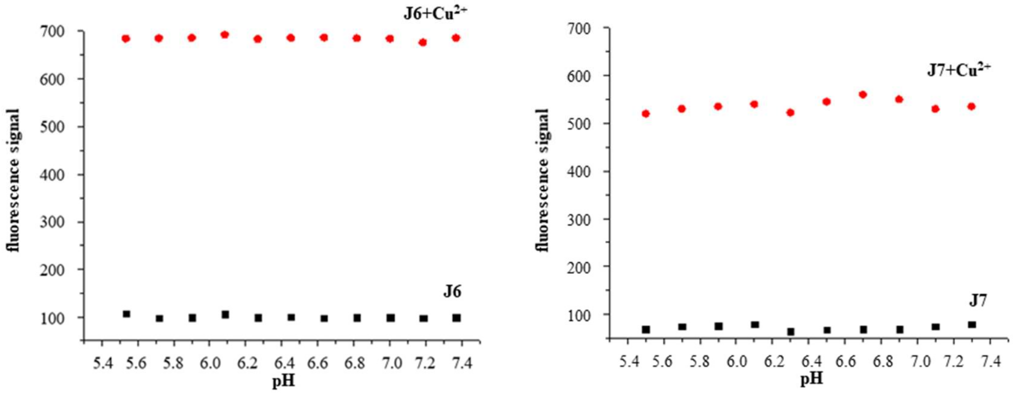

Figure S1. Influence of pH on **J6**/**J7** (8 μmol L^{-1}) in the absence and presence of Cu^{2+} (8 μmol L^{-1}).

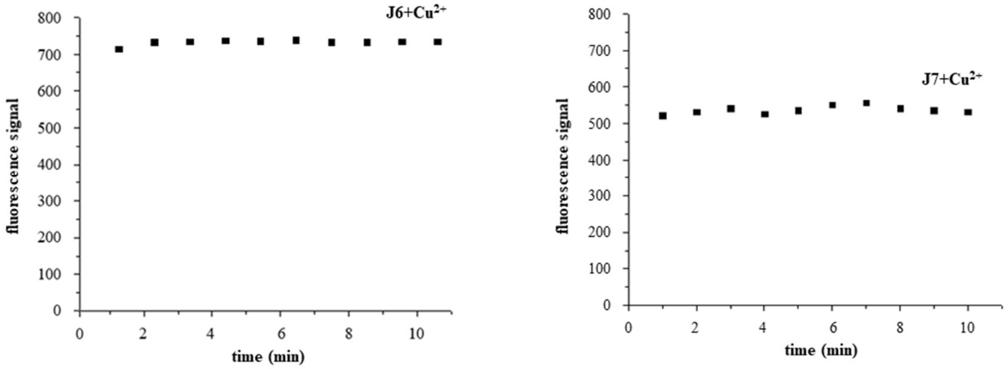

Figure S2. Influence of time on **J6**/**J7**-copper ions system (probe: 8 μmol L^{-1}, Cu^{2+}: 8 μmol L^{-1}).

Table S1. Cytotoxicity of probes **J6** and **J7** in ECV304 at 24 h.

**concentration (μg L^{-1})**	**cytotoxicity (%)**	
	**J6**	**J7**
125	0.08±0.07	0.61±0.21
250	1.10±0.54	0.96±0.29
500	3.45±0.67	3.40±0.68
1000	7.43±1.39	14.30±2.23
2000	10.49±1.30	8.20±1.43
4000	27.90±2.49	13.00±1.69

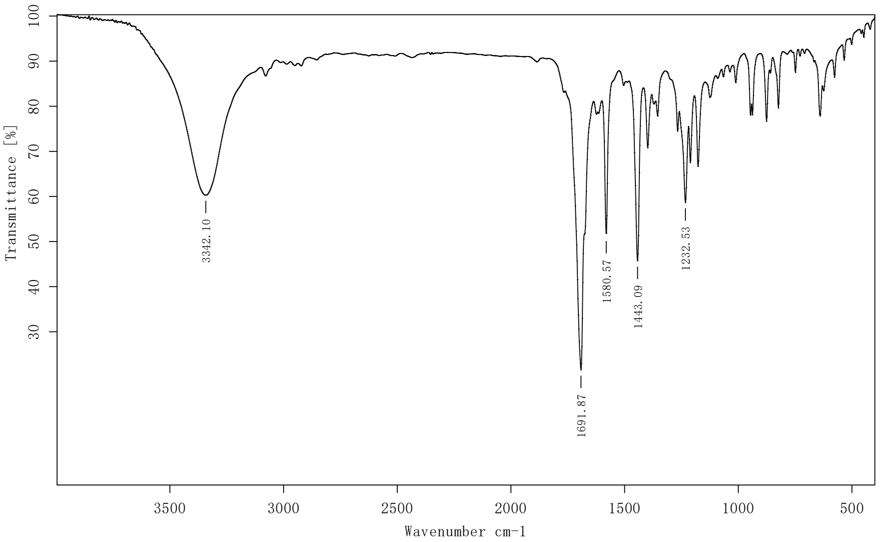

Figure S3. IR of 6-hydroxyl-4-methylcoumarin (a).

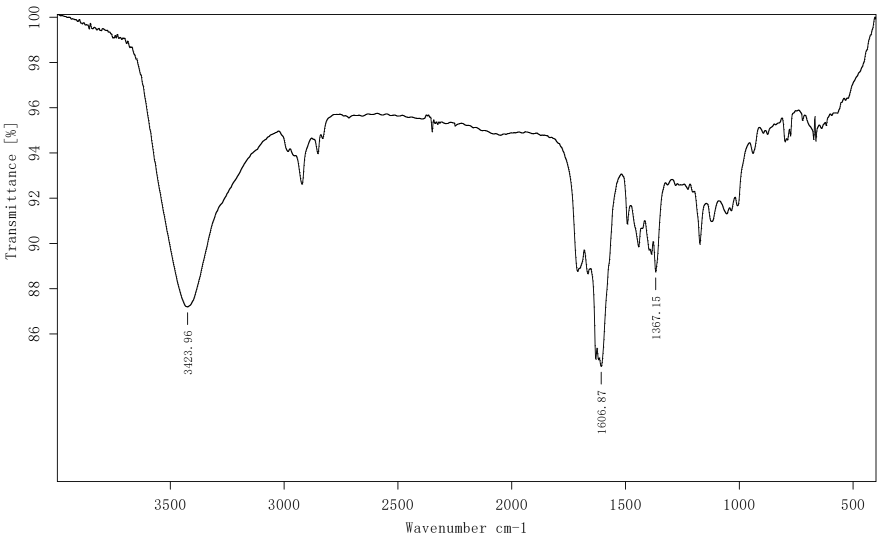

Figure S4. IR of 5-formyl-6-hydroxyl-4-methylcoumarin (b).

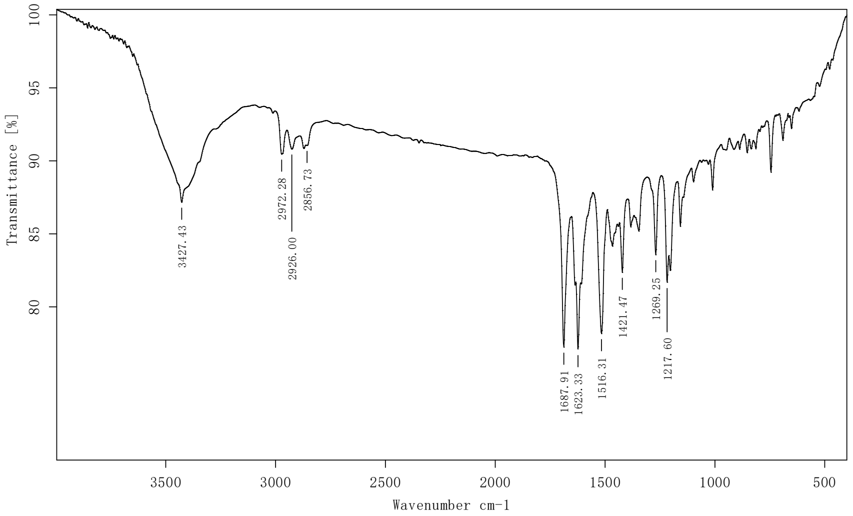

Figure S5. IR of rhodamine 6G acylhydrazine (c).

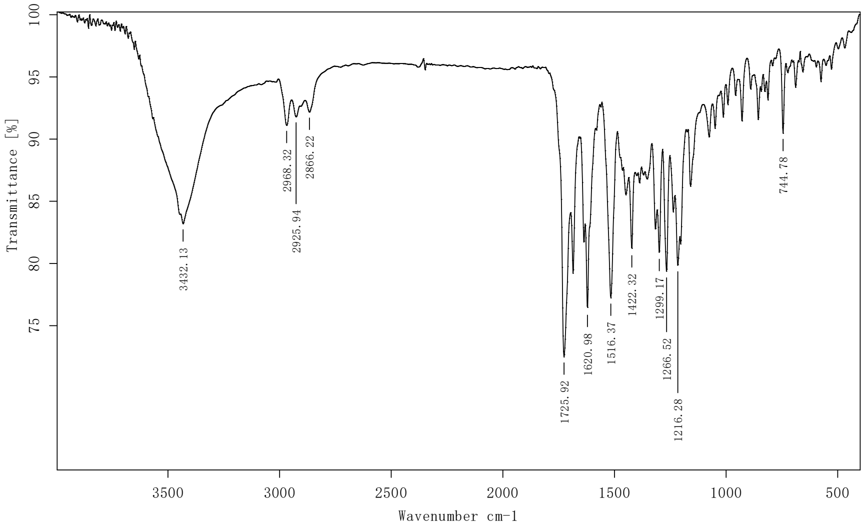

Figure S6. IR of probe **J6**.

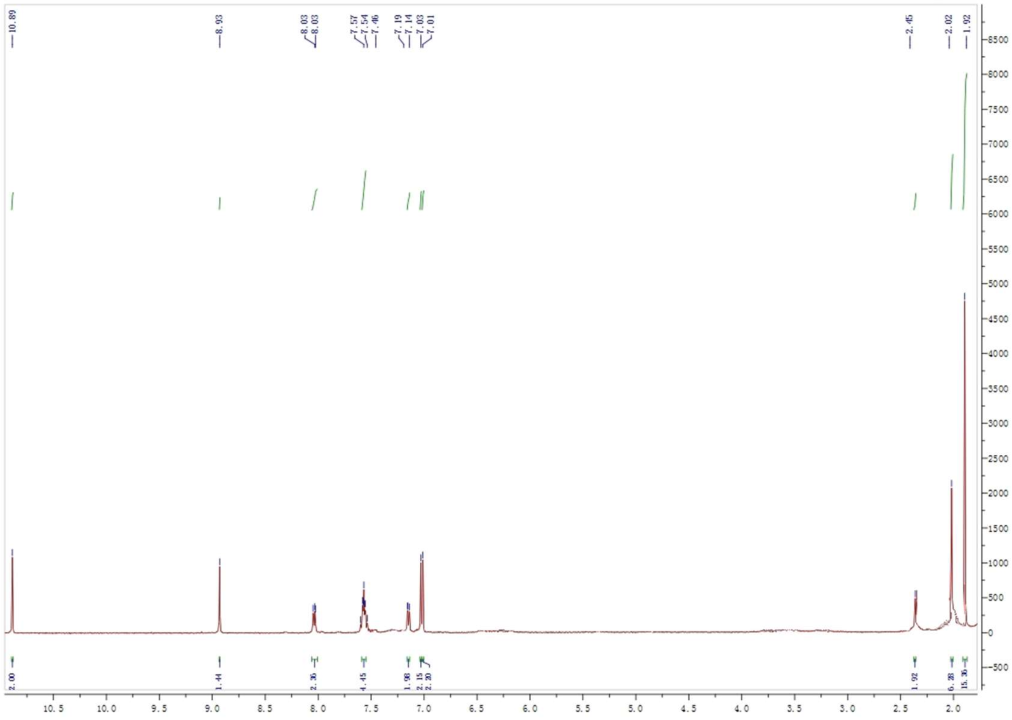

Figure S7. ^{1}H NMR of probe **J6**.

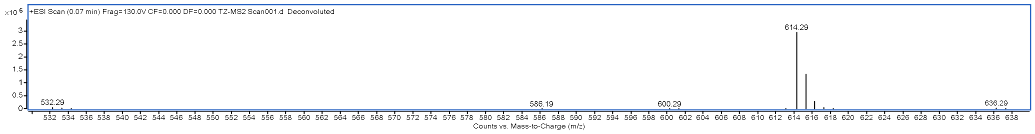

Figure S8. MS of probe **J6**.

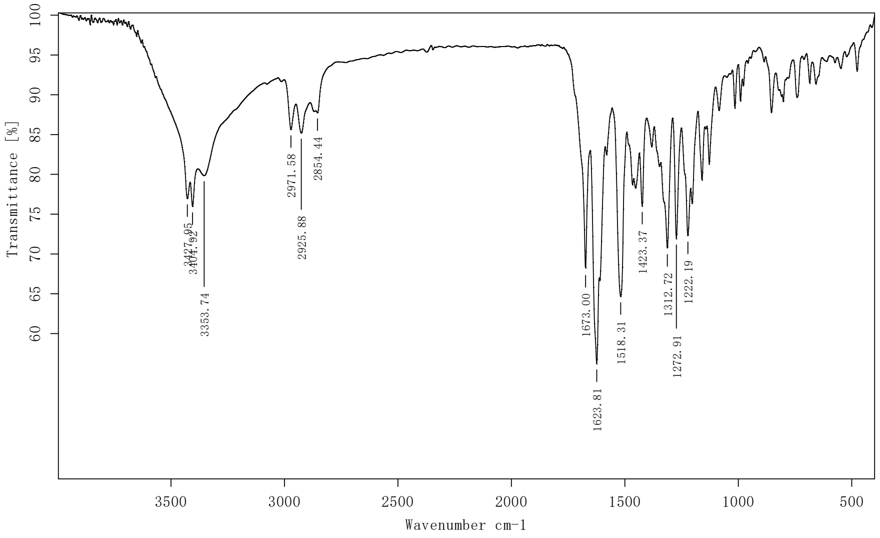

Figure S9. IR of probe **J7**.

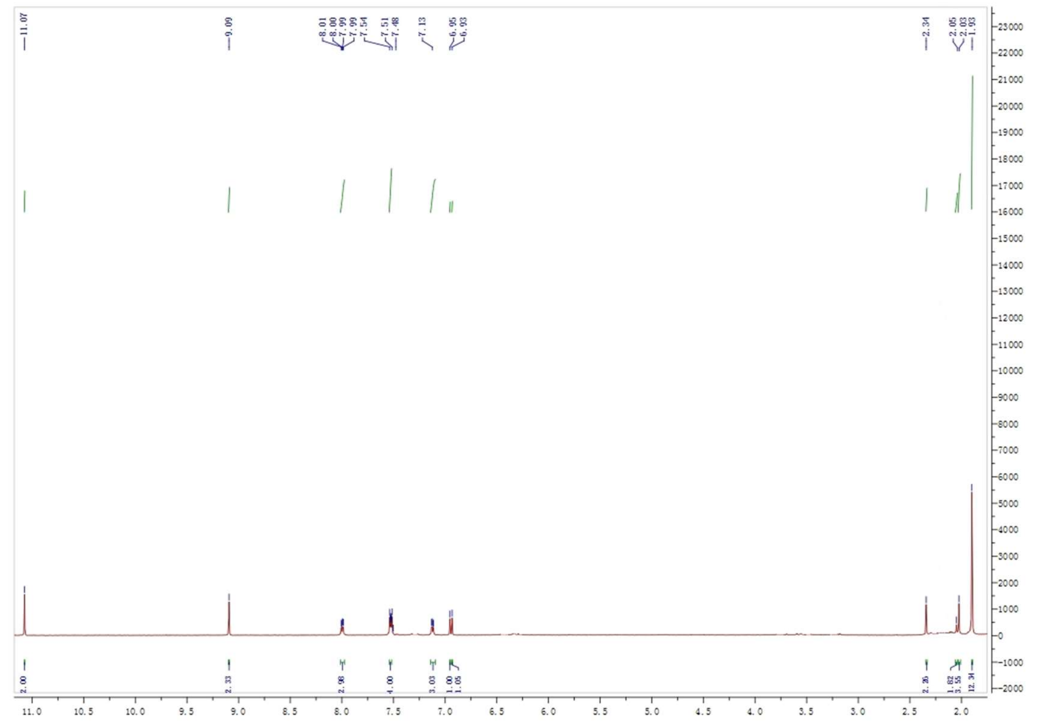

Figure S10. ^{1}H NMR of probe **J7**.

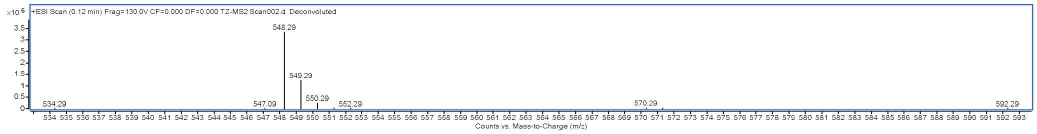

Figure S11. MS of probe **J7**.
